# Age-related changes in dentate gyrus cell numbers, neurogenesis, and associations with cognitive impairments in the rhesus monkey

**DOI:** 10.3389/fnsys.2015.00102

**Published:** 2015-07-14

**Authors:** Laura B. Ngwenya, Nadine C. Heyworth, Yamin Shwe, Tara L. Moore, Douglas L. Rosene

**Affiliations:** ^1^Department of Anatomy and Neurobiology, Boston University School of Medicine, Boston, MAUSA; ^2^Yerkes National Primate Research Center, Emory University, Atlanta, GAUSA

**Keywords:** adult neurogenesis, dentate gyrus, bromodeoxyuridine, doublecortin, non-human primate, stereology, learning, cognitive aging

## Abstract

The generation of new neurons in the adult mammalian brain is well-established for the hippocampal dentate gyrus (DG). However, the role of neurogenesis in hippocampal function and cognition, how it changes in aging, and the mechanisms underlying this are yet to be elucidated in the monkey brain. To address this, we investigated adult neurogenesis in the DG of 42 rhesus monkeys (39 cognitively tested) ranging in age from young adult to the elderly. We report here that there is an age-related decline in proliferation and a delayed development of adult neuronal phenotype. Additionally, we show that many of the new neurons survive throughout the lifetime of the animal and may contribute to a modest increase in total neuron number in the granule cell layer of the DG over the adult life span. Lastly, we find that measures of decreased adult neurogenesis are only modestly predictive of age-related cognitive impairment.

## Introduction

The hippocampus is critical for cognitive functions, particularly for the processes of learning and memory (Squire, 1992). Early studies on the functional role of the hippocampus were based on the classical case of patient H.M. who developed a severe anterograde amnesia following the bilateral removal of the medial temporal lobe to reduce epileptic seizures (Scoville and Milner, 1957). Further neuropsychological assessments on H.M. demonstrated that the medial temporal lobe was necessary specifically for declarative memory formation, while other intellectual functions remained largely intact (Milner, 1966, 1972; Corkin, 1984).

While the medial temporal lobe contains other anatomical structures that were damaged in H.M.’s surgery (Corkin et al., 1997), data from other studies (Penfield and Milner, 1958) and other domains (e.g., Alzheimer’s) continue to point to components of the hippocampal formation as critical for declarative and episodic memory. Within the hippocampal formation, information is processed through the classic “trisynaptic circuit.” The DG stands at the start of this circuit receiving, via the perforant path, cortical inputs from the entorhinal cortex (EC; McNaughton and Morris, 1987). The medial EC provides spatial information, and the lateral EC conveys non-spatial information (Hargreaves et al., 2005; van Strien et al., 2009). Mossy fibers from the DG project to the CA3 subfield of the hippocampus, where the Schaffer collaterals then project to the CA1 subfield. Output from the hippocampus consists of CA1 projections both directly to cortical and subcortical targets, as well as to the adjacent Subiculum, which gives rise to cortical and subcortical projections (Morris, 2006). Hence, the DG stands as the gateway into this trisynaptic circuit.

Studies examining the functional role of the DG in rodents have demonstrated the DG (specifically the dorsal DG, dDG) to be critical for conjunctive encoding of multiple sensory inputs, spatial and contextual pattern separation, and temporal integration of remote memory (for review, see Kesner, 2013). Pattern separation, which involves the ability to distinguish cues when they are spatially and temporally similar, has been shown to be the keystone of hippocampal learning, important for recollection and episodic memory. This has been elucidated by lesion studies in rodents. Gilbert et al. (2001) revealed that rats with dDG lesions were significantly impaired at short spatial separations when performing a pattern separation task. In another study using a place preference procedure in an eight-arm maze, rats with dDG lesions required significantly more trials in spatially adjacent conditions, again demonstrating the importance of the dDG in spatial pattern separation (Morris et al., 2012). It is thought that the DG is necessary for pattern separation, and that the DG to CA3 connection plays a crucial role in this feature of hippocampal function.

Much less is known about the specific hippocampal subfield functions in humans and NHPs, although studies have reported that the DG may also be important for pattern separation in primates. Studies in humans using high-resolution functional magnetic resonance imaging have reported that the CA3/DG regions play a role in pattern separation (Bakker et al., 2008). Similarly, also using functional MRI, Yassa et al. (2011) demonstrated a functional deficit in CA3/DG activity in older adults compared with young adults during a pattern separation task. These results reflect similar findings to rodent lesion studies, requiring more highly dissimilar items for successful differentiation between previously identified items.

A unique feature of the DG is the presence of adult neurogenesis in the subgranular zone (SGZ); adult generated cells differentiate into granule neurons of the DG. Since the first evidence of adult neurogenesis in the postnatal rat hippocampus by Altman and Das (1965), adult neurogenesis has been well-characterized in the DG of rodents (Kaplan and Hinds, 1977; Kuhn et al., 1996; Kempermann et al., 1997; Cameron and McKay, 2001), with more recent studies investigating the functional role of adult-generated neurons. For example, electrophysiological studies of adult neurogenesis have demonstrated that adult-born neurons are first functionally silent, but become hyperexcitable during the immature phase. Once a mature phenotype is expressed, they are physiologically indistinguishable from surrounding granule cells (Aimone et al., 2010). Other investigations of the functional contribution of adult neurogenesis have used low-dose irradiation to arrest production of newborn neurons in the DG of mice and shown that pattern separation tasks are impaired (Clelland et al., 2009). Transgenic mouse models have also been used to transiently reduce the number of adult-born neurons in a temporally regulated manner. Deng et al. (2009) used this approach in mice with a conditional knockout on the nestin promoter, to demonstrate that a reduced number of immature neurons impaired performance on the Morris Water Maze task and in contextual fear extinction. Overall, these findings suggest that adult-born neurons contribute to hippocampal-dependent cognitive tasks.

While these findings in rodents raise the intriguing prospect that adult neurogenesis plays an important functional role in hippocampal function, we know much less about these processes in human and NHPs. A seminal study by Eriksson et al. (1998) first demonstrated the existence of human adult hippocampal neurogenesis. Since then, additional human studies have provided supplemental evidence that newly generated neurons are present in the hippocampus (Knoth et al., 2010; Spalding et al., 2013). In the NHP, adult neurogenesis has been shown to occur in the hippocampus (Gould et al., 1999; Kornack and Rakic, 1999; Ngwenya et al., 2006). Similar to rodents, adult generated neurons in the NHP undergo a maturation process that recapitulates that seen in development (Ngwenya et al., 2006). However, in rodents, the maturation process for new neurons takes ∼1 month, whereas in primates, it has been demonstrated that the maturation process can take in excess of three (Ngwenya et al., 2006), and potentially as long as 6 months (Kohler et al., 2011).

Another critical question regarding adult neurogenesis is how the process changes across the adult life span. In rodents, it has been shown that adult neurogenesis in the hippocampus persists into old age but undergoes progressive declines (Kuhn et al., 1996; Seki, 2002; McDonald and Wojtowicz, 2005). In NHPs, studies that include aged animals also demonstrate the presence of an age-related decline in neurogenesis (Gould et al., 1999; Leuner et al., 2007; Aizawa et al., 2011). Furthermore, studies in humans have demonstrated hippocampal neurogenesis also persists into old age along with an age-related decline (Knoth et al., 2010). Recent carbon dating of hippocampal cells in human brain has shown that the decline in turnover with aging is fourfold between 16 and 91 years of age (Spalding et al., 2013), compared to published rodent data showing a 10-fold decline in neurogenesis from 2 to 9 months of age in mice (Ben Abdallah et al., 2010). In primates, the details of the decline in neurogenesis with aging and the longevity of newly generated neurons have not been fully elucidated.

As adult neurogenesis decreases exponentially with age, attention has been focused on the role of adult neurogenesis in age-dependent cognitive functions. In monkeys, age-related declines in cognitive function are well-documented, but the cause is not entirely clear. Since monkeys do not show Alzheimer’s disease pathology and cortical neurons are not significantly lost with aging, the search for other substrates has focused on white matter pathology (Peters et al., 1996) and on cortical neurophysiology (Luebke et al., 2004). While both age-related pathologies correlate with various aspects of cognitive decline, the possible contribution of declining neurogenesis in the DG is unknown. This field of research has been encouraged by rodent studies showing that aging results in a decline of neural progenitor cells and neurogenesis with correlations with cognitive function (Drapeau et al., 2003; van Praag et al., 2005). However, the current data are not consistent with a potential casual link as other studies have demonstrated no relationship, or inverse relationships between cell proliferation or adult neurogenesis and cognitive performance (Bizon and Gallagher, 2003; Gallagher et al., 2003; Merrill et al., 2003).

Here, we address the questions of declining neurogenesis, maturation and longevity of newly created neurons, and relationship to cognitive function with aging in a cohort of 42 behaviorally tested rhesus monkeys. Specifically, we used animals of both sexes and ages that cover the adult life span ranging from 6.1 to 31.5 years of age (equivalent to 18 to 93 years-old humans). To evaluate cell proliferation, we used the endogenous marker Ki-67 to identify cells in the cell cycle, and the exogenous marker BrdU to label cells during S-phase (Gratzner, 1982; Gerdes et al., 1984; Miller and Nowakowski, 1988; Cameron and McKay, 2001; Kee et al., 2002). To assess the long-term survival of adult generated neurons, we varied the interval between BrdU injection and tissue harvest from 3 weeks to 1.5 years. Although BrdU and Ki-67 both provide information on proliferative capacity they do not distinguish between adult generated neurons and non-neuronal cells such as glia. To identify cells in the neuronal lineage we used antibodies to the protein DCX, which is transiently expressed in newly generated neurons and has been used as a marker of adult neurogenesis (Francis et al., 1999; Gleeson et al., 1999; Brown et al., 2003; Rao and Shetty, 2004; Couillard-Despres et al., 2005). To assess the maturation and survival of adult generated neurons in animals of varying ages, we evaluated the double-labeling of BrdU with either DCX or the mature neuronal marker NeuN (Mullen et al., 1992). Additionally, we used stereological methods to evaluate quantitative changes in number and volume of the DG with age. Here, we show that adult neurogenesis persists in aging NHP but demonstrates an age-related decline, new neurons are additive to the DG, and that neurogenesis is not the sole predictor of cognitive decline with aging.

## Materials and Methods

### Subjects

A total of 42 adult rhesus monkeys (*Macaca mulatta*), ranging in age from 6.1 to 31.5 years, were used in this study. Both males and females were included, but stereological analyses were conducted only on male subjects to decrease variability. Most animals were part of a larger cohort of monkeys used to study the effects of normal aging on cognitive function (P01-AG000001) and so were part of multiple other investigations. The distribution of all subjects across different experiments is outlined in **Table 1**. Monkeys were obtained from Yerkes National Primate Research Center (Atlanta, GA, USA), Labs of Virginia (Yemassee, SC, USA), or Covance Laboratory (Princeton, NJ, USA). For approximately the last year of the animals’ life, they were individually housed in a 12 h light–dark cycle in the Laboratory of Animal Science Care at Boston University School of Medicine. This facility is accredited by the Association for the Assessment and Accreditation of Laboratory Animal Care and is staffed by licensed veterinarians and trained primate care staff. All procedures followed National Institutes of Health guidelines and were approved by Boston University Medical Campus Institutional Animal Care and Use Committee.

**Table 1:** All subjects – ordered by age.

Animal ID	Age	Sex	BrdU survival time (weeks)	BrdU stereology	Ki-67 stereology	DCX stereology	Doubl label IHC	Hippocampal stereology	Cognitive testing
AM010	6.1	M						X	
AM205	6.3	M						X	X
AM247	6.9	M	3	X		X	X		
AM245	7.0	M	3	X		X	X		
AM132	7.5	M	3	X		X	X		X
AM093	7.6	M						X	X
AM128	7.9	M	3	X	X	X	X	X	X
AM280	8.1	M	38				X		X
AM229	8.3	M	14				X		X
AM047	9.0	M						X	X
AM289	9.0	M	28				X		X
SM003	9.2	M	82				X		X
AM255	9.5	F	72				X		X
AM053	9.6	M						X	X
AM202	10.4	F			X				X
AM254	11.4	F	48				X		X
AM137	12.6	M	3	X				X	X
AM136	12.6	M	3	X	X	X			X
AM144	14.9	M	3	X		X		X	X
AM143	15.8	M	3	X		X		X	X
AM237	17.6	M	43				X		X
AM250	18.0	F	61				X		X
AM190	18.1	F			X				X
AM253	18.4	F	57				X		X
AM153	19.5	M	3	X	X	X	X	X	X
AM133	19.5	M	3	X		X		X	X
AM159	19.8	F			X				X
AM149	19.8	F			X				X
AM124	19.9	M	3	X		X	X	X	X
AM252	19.9	F	83				X		X
AM256	20.7	F	78				X		X
AM208	22.7	M	3	X	X	X	X	X	X
AM236	22.9	M	47				X		X
AM242	23.5	M	81				X		X
AM179	23.7	F			X				X
AM189	24.5	M	3	X		X	X	X	X
AM070	25.4	M						X	X
AM110	25.8	M			X			X	X
AM283	26.4	M	23				X		X
AM027	27.9	M						X	X
AM180	29.6	F			X				X
AM091	31.5	M						X	X

### BrdU Administration

Twenty-six animals received a single intraperitoneal injection of 200 mg/kg of BrdU (Sigma, St. Louis, MO, USA), prepared at a concentration of 15 mg/ml, in sterile Tris-buffered saline at pH 7.4. The majority of the animals were perfused 3 weeks after BrdU injection (**Table 2**), but others that were part of a long-term survival study were perfused at varying times after BrdU injection, as summarized in **Table 3**.

**Table 2:** Males with 3 weeks BrdU survival time – ordered by age.

Animal ID	Age (years)	Sex	BrdU Surv time (weeks)	BrdU stereology	Ki-67	DCX stereology	Fluorescence IHC	Hippocampal stereology	Cognitive testing
AM247	6.9	M	3	X		X	X		
AM245	7.0	M	3	X		X	X		
AM132	7.5	M	3	X		X	X		X
AM128	7.9	M	3	X	X	X	X	X	X
AM137*	12.6	M	3	X				X	X
AM136	12.6	M	3	X	X	X			X
AM144	14.9	M	3	X		X		X	X
AM143	15.8	M	3	X		X		X	X
AM153	19.5	M	3	X	X	X	X	X	X
AM133	19.5	M	3	X		X		X	X
AM124	19.9	M	3	X		X	X	X	X
AM208	22.7	M	3	X	X	X	X	X	X
AM189	24.5	M	3	X		X	X	X	X

*Indicates animal that does not have CSST behavioral measures.

**Table 3:** Animals used for fluorescence IHC – ordered by BrdU survival.

Animal ID	Age (years)	Sex	BrdU Surv time (weeks)	Cognitive testing	%BrdU+DCX+ in GCL	%BrdU+NeuN + in GCL
AM247	6.9	M	3		37	
AM245	7.0	M	3		31	
AM132	7.5	M	3	X	13	
AM128	7.9	M	3	X	20	
AM153	19.5	M	3	X	0	
AM124	19.9	M	3	X	0	
AM208	22.7	M	3	X	0	
AM189	24.5	M	3	X	0	
AM229	8.3	M	14	X	22	
AM283	26.4	M	23	X	33	
AM289	9.0	M	28	X	11	
AM280	8.1	M	38	X	5	
AM237	17.6	M	43	X	0	60
AM236	22.9	M	47	X		13
AM254	11.4	F	48	X		48
AM253	18.4	F	57	X		57
AM250	18.0	F	61	X		57
AM255	9.5	F	72	X		52
AM256	20.7	F	78	X		11
AM242	23.5	M	81	X		21
SM003	9.2	M	82	X		66
AM252	19.9	F	83	X		55

### Tissue Acquisition

At the conclusion of the experiment, animals were deeply anesthetized and the brain was fixed by transcardial perfusion with either 4% paraformaldehyde, or 1% paraformaldehyde and 1.25% glutaraldehyde as previously described (Ngwenya et al., 2005; Giannaris and Rosene, 2012). Brains were cryoprotected, flash-frozen, and stored at -80°C until processed (Rosene et al., 1986). One hemisphere per animal was sectioned in the coronal plate into ten interrupted series, one of which was slide mounted and Nissl stained with 0.05% thionin. Other series were placed into cryoprotectant and stored at -80°C until batch processed for immunohistochemistry as described below and in detail by Giannaris and Rosene (2012).

### Batch Processing and Immunohistochemistry

For batch processing, series of cut sections from all relevant subjects were removed together from -80°C storage, thawed at room temperature and rinsed in buffer. All sections were processed together using the same batch of reagents to minimize the possibility of procedural variance. The number of tissue sections used was determined by pilot experiments to calculate the average number of sections necessary for examination of the minimum number of cells.

Immunohistochemistry protocols were optimized for each reagent as in Hoffman et al. (2008). For diaminobenzidine (DAB) immunohistochemistry, all sections were quenched of peroxidases, blocked in serum, then incubated in appropriate primary antibody overnight. Sections were washed, incubated in biotinylated secondary antibody and treated with a biotin–avidin complex (Vector Elite Kit; Vector Labs, Burlingame, CA, USA). They were then incubated with (Sigma) and developed with H_2_O_2_ (1%). Sections were subsequently mounted onto glass slides and coverslipped.

For Ki-67 immunohistochemistry, a series of every 10th section spaced at 300 μM throughout the hippocampus from 11 selected cases were batch processed to assess cell proliferation. Primary antibody incubation was for 48 h with mouse anti-Ki-67 (1:200; Vector Labs).

For BrdU immunohistochemistry, a series of every 20th section spaced at 600 μM throughout the entirety of the hippocampus from 13 cases were processed as previously described (Ngwenya et al., 2005). Sections were pretreated with 2N HCl to allow access to the antigen, and tissue was incubated with mouse anti-BrdU primary antibody (1:200; Chemicon, Temecula, CA, USA).

For DCX immunohistochemistry, a series of every 40th section spaced at 1200 μm throughout the hippocampus from 12 cases were used and sections were post-fixed in 4% Paraformaldehyde + 2.5% Acrolein for 10 min, rinsed for 20 min in sodium borohydride, and then incubated in primary antibody (rabbit anti-DCX; 1:10,000; Cell Signaling, Danvers, MA, USA) for 72 h.

### Stereological Estimations of DAB Labeled Cells

Labeled cells were identified using a 60x objective on a Nikon E600 series microscope (Nikon; Melville, NY, USA), equipped with a motorized stage, and controlled by StereoInvestigator software (MicroBrightField; Williston, VT, USA). The estimated total number of cells was obtained for the granule cell layer (GCL) and hilus with the operator blind to subject age and cognitive ability using an exhaustive sampling scheme as previously described (Ngwenya et al., 2005). Numbers (N) were calculated using the optical fractionator where N = Q x 1/asf x 1/ssf x 1/tsf, as in West et al. (1991). This counting scheme was used for the estimated total number of Ki-67, BrdU, and DCX labeled cells.

### Double-Label Immunofluorescence for BrdU and Maturation Markers

For BrdU and NeuN immunohistochemistry, a series of every 10th section spaced at 300 μm throughout the hippocampus from 10 cases with BrdU survival times ranging from 43 to 83 weeks was processed for double-labeling (**Table 3**). For primary incubation, tissue was treated with a pooled solution containing antibodies for BrdU (rat anti-BrdU;1:200; Accurate Chemical, Westbury, NY, USA) and NeuN (mouse anti-NeuN; 1:200; Chemicon) for 72 h. Sections were then washed, incubated with pooled highly cross-adsorbed secondary antibodies (anti-rat Alexa Fluor 488; and anti-mouse Alexa Fluor 568, both 1:250; Life Technologies, Grand Island, NY, USA). Sections were then mounted onto slides and coverslipped with PVA-DABCO (Sigma). For BrdU and DCX immunohistochemistry, a series of every 20th section spaced at 600 μM throughout the entirety of the hippocampus from 13 cases with BrdU survival times ranging from 3 to 43 weeks were processed for double-labeling (**Table 3**). Batch processing for BrdU and DCX immunofluorescence was conducted using a sequential protocol rather than pooled antibody solution as the DCX signal is less robust in that age range. Sections were first processed for BrdU with incubation in primary antibody (mouse anti-BrdU, 1:200; Chemicon), then incubation in secondary antibody (anti-mouse Alexa Fluor 488; 1:200; Life Technologies). Tissue processing continued for DCX with incubation in primary antibody (rabbit anti-DCX; 1:300; Cell Signaling) and secondary antibody (anti-rabbit Alexa Fluor 568; 1:1000; Life Technologies). Finally, tissue was processed with Autofluorescence Eliminator (Millipore, Billerica, MA, USA), mounted onto slides and coverslipped with PVA-DABCO (Sigma; St. Louis, MO, USA).

### Confocal Analysis of Double Label Immunofluroescence

Confirmation of double labeling for BrdU-NeuN and BrdU-DCX was performed using optimal slice z-stack analysis on a Zeiss Laser Scanning Confocal Microscope (Zeiss LSM710; Carl Zeiss Microimaging). For each set of double stained sections, the percentage of double-labeled cells was calculated by examining the number of BrdU labeled cells that was double labeled with the neuronal marker, divided by the total number of BrdU labeled cells present (both double and single labeled). In animals with long times from BrdU administration to tissue harvest, BrdU labeled cells were often sparse. A minimum of 20 BrdU positive cells was required per animal to be included in analysis of double labeling.

### Stereological Analysis of Hippocampal Volumes and Granule Cell Numbers

A series of every fifth Nissl stained section spaced at 1500 μm throughout the hippocampal formation from 18 male rhesus monkeys were analyzed for stereologic estimation of total neuron number. Sections were blind coded and counted using the optical fractionator method (West et al., 1991) with a 100x objective on a Nikon E600 microscope equipped with a Q-Imaging digital camera and ASI MS-2000 motorized stage controlled by Bioquant Stereology Toolkit (version 6.75.10; Bioquant Image Analysis Corporation, Nashville, TN, USA). Hippocampal ROI were outlined using a 2x objective. Then neurons in the GCL were counted using the 100x oil immersion objective with the granule cell nucleolus as the counting object. A sampling grid size of 300 μm × 300 μm was placed randomly over the granule cell ROI to position the 10 μm × 10 μm counting frame in xy with a 1 μm z-axis guard volume. Nucleoli in the GCL were only counted when the nucleus, and at least a portion of the cell body to which the nucleolus belonged, could be identified. This assured that nucleoli of glial nuclei that may reside in the GCL were not counted, and additionally assured that each neuron was only counted once. Neurons with multiple nucleoli were not observed. Similar to BrdU stereology, the estimated total number of cells was calculated using N = Q x 1/asf x 1/ssf x 1/tsf, as in West et al. (1991). Volume estimates were obtained by planimetry and by the Cavalieri principle utilizing V_ref_ = T x ΣA_1-n_ (Gundersen and Jensen, 1987) for the total DG and for the remainder of the hippocampus (that volume which excluded the DG). The coefficient of error was calculated as in Gundersen et al. (1999) and was 0.1 or less for all cell counts and volume measurements.

### Cognitive Testing

Thirty-nine of the 42 animals were assessed on tests of learning, memory and executive function, including DNMS, DRST, and CSST. DNMS and DRST were assessed in a Wisconsin General Testing Apparatus and CSST was assessed in an automated testing box. These tasks have been detailed in previous studies and are described briefly below (Moss, 1993; Herndon et al., 1997; Moore et al., 2005).

#### DNMS Rule Learning

The DNMS task is a benchmark recognition memory task that assesses the subject’s ability to distinguish a novel from a familiar stimulus after a specific delay interval (Herndon et al., 1997). The acquisition portion of DNMS assesses rule learning and was conducted by placing a reinforcer, such as an M&M/raisin/peanut, in the center well of a testing board under a sample object. The monkey was required to displace the sample object to obtain the reinforcer. The sample object was then placed over one of the two lateral (unbaited) wells, and a new, unfamiliar object was placed over the other lateral well which was baited with a reinforcer. After a 10 s interval, the monkey was given a recognition trial to choose between the two objects and was rewarded for choosing the unfamiliar, novel object. Testing continued with 10 trials a day until criterion for learning this non-match rule was accomplished by the monkey completing 90 correct responses in 100 consecutive trials. Different forms of the DNMS task have been used to assess memory function in monkeys following specific lesions within the temporal lobe limbic system (Gaffan, 1974; Mahut et al., 1982; Alvarez et al., 1995).

#### DNMS Recognition Memory

Following acquisition of the DNMS non-match rule, the delay interval between the sample presentation and the recognition choice was increased from 10 s to 2 min to measure memory over this delay. After 100 trials with the 2 min delay (10 trials per day for 10 days), the monkey was tested for another 100 trials (10 trials per day for 10 days) with a 10 min delay.

#### DRST Working Memory Capacity

Following DNMS testing, monkeys were tested on DRST to assess short-term working memory capacity in two different domains – spatial memory and object memory. Testing in the spatial domain consisted of baiting one well in an 18 well-board (3 × 6) with a reinforcer and then covering the baited well with a plain disk. The monkey then displaced the disk to retrieve the reward. A door was then lowered and the first disk was placed back in the initial location and a new well, in a pseudo random location was baited and covered with a second identical disk. The door was then raised and the monkey was rewarded for identifying the new location and displacing the second disk. If an error was made, the trial was ended and a span of 1 was recorded. If the trial was successful, then both of the first and second disks were returned to their original locations and a new well was baited and covered with a third disk. The monkey was then allowed to obtain a reward by displacing the new disk based on its novel location. Trials continued in this way until the monkey made an error, which ended the trial and the span score was determined by the total number of disks the animal identified correctly by novel spatial location. Each day a total of 10 trials were presented. Testing continued at 10 trials per day until 100 trials were completed. The next day the DRST object test was initiated. While similar to the spatial condition, in this case wells were covered with unique objects rather than identical disks and spatial location was randomized for all stages. Thus after the monkey displaced the first object and obtained the reinforcer, the first object was placed over a randomly located, unbaited well and a second well was baited and covered with a novel object. The monkey was rewarded for choosing the novel object. This procedure of repositioning all previously presented objects on the testing board while adding a new object over the next baited well-continued until the monkey made an error by failing to choose the novel, baited object. Again a total of 100 trials were administered at 10 trials per day. DRST was originally designed to investigate recognition memory in monkeys following bilateral removal of the hippocampus, but has also been used as an assessment of recognition memory in human populations (Moss et al., 1986; Inouye et al., 1993).

#### CSST Assessment of Executive Function

The CSST is modified from the Wisconsin Card Sorting Task (WCST), which is used as an assessment of executive function in humans. CSST was administered in an automated testing apparatus (Moore et al., 2005). Animals were first trained to touch one of nine locations on a touch screen to get a reward. Subsequently, on each trial, three different objects that differed in color (red, green, or blue) and shape (triangle, star, or square) appeared in random locations. The initial test required the subject to learn to touch a red object, regardless of its shape, to receive a reward. Animals were given 80 trials per day, with an inter-trial interval of 15 s. Once the animal reached a criterion of 10 consecutive correct trials, the reward criterion shifted to a new category, the shape of triangle, which was rewarded regardless of color. The test continued in this manner until the criterion of 10 correct responses was reached and then the category switched to the reward contingency of blue regardless of shape. The final switch was then to star regardless of color. The number of errors to criterion was recorded for each category. After a switch occurred, the number of errors in which the animal continued to choose the previously rewarded concept was recorded as perseverative errors. When an animal made six to nine correct responses, but then made an error prior to reaching criterion of 10 correct responses, a broken set was recorded.

### Statistical Analysis

All statistics were done using SPSS v20.0 for Macintosh (SPSS, Inc., Chicago, IL, USA). Initial analysis of all data included confirmation that the relationships between parameters were linear, and that the data did not violate the assumptions of linearity, equal variances, or normality. Linear and stepwise multiple regression was done utilizing a least-squares-fit approach and two-tailed statistics with significance defined at a *p*-value of less than 0.05.

## Results

### Proliferative Capacity Decreases in the Aging Monkey

**FIGURE 1:**
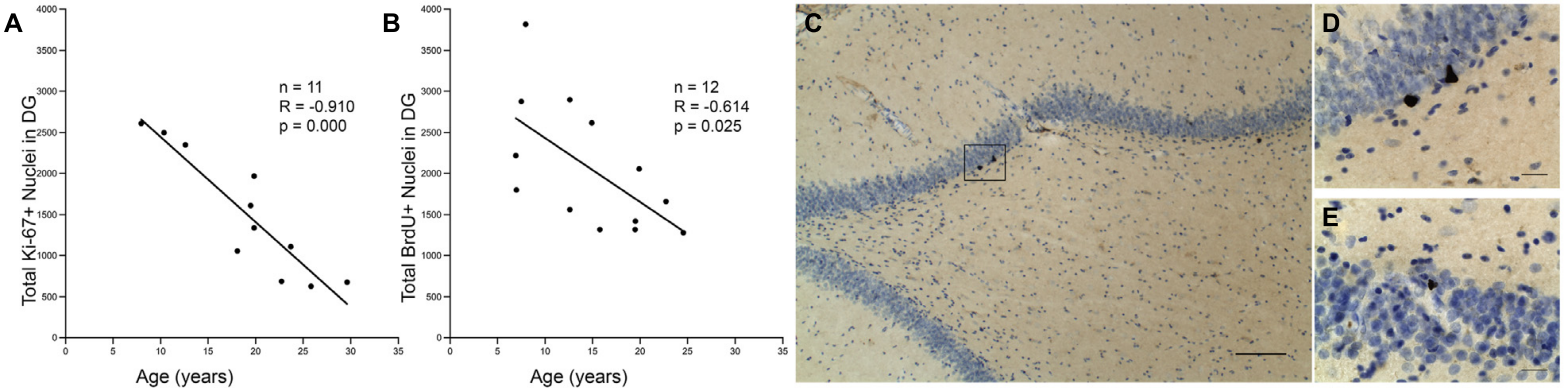
**Capacity for neurogenesis declines with age. (A)** The total number of Ki-67 positive nuclei significantly declines with age. Regression analysis predicts a 68% decline in Ki-67 positive cells between a 7 and a 25-years-old monkey (threefold change). **(B)** The total number of BrdU positive cell nuclei that are present after a 3-weeks survival also shows a significant negative correlation with age. Regression predicts a 53% decline between ages 7 and 25, which corresponds to a twofold change in BrdU labeled cells. **(C)** A photomicrograph illustrates BrdU immunohistochemistry with cresyl violet counterstain in the DG of a young monkey; scale bar = 100μm. The box represents a cluster of BrdU positive nuclei, which is enlarged in **(D)**. **(E)** Aged animals also show clusters of BrdU positive nuclei as shown here. Scale bar for **(D,E)** = 20 μm.

Eleven monkeys, aged 7.9–29.6, were processed for Ki-67 immunohistochemistry to identify the number of cells in the cell cycle as an assay of proliferative activity in the DG (**Table 1**). The total number of Ki-67 positive cells in the DG was quantified using stereology. As shown in **Figure 1A**, Pearson’s correlation showed a significant decrease in proliferative capacity with age with *r* = -0.910, *p* < 0.01. Thirteen monkeys, aged 6.9–24.5 (**Table 1**), were injected with a single injection of BrdU 3 weeks before perfusion-fixation of the brain. As shown in **Figure 1B**, stereological counts of the number of BrdU+ cells in the DG revealed a significant decrease in the number of BrdU+ cells with age (*r* = -0.614, *p* = 0.025). Together, these results demonstrate that both the total proliferative capacity and 3-weeks survival of adult generated cells in the DG decline significantly with age. Nevertheless, it is worth noting that proliferative cells were detected even in the oldest animals examined.

### Immature Neuron Production Declines with Age

**FIGURE 2:**
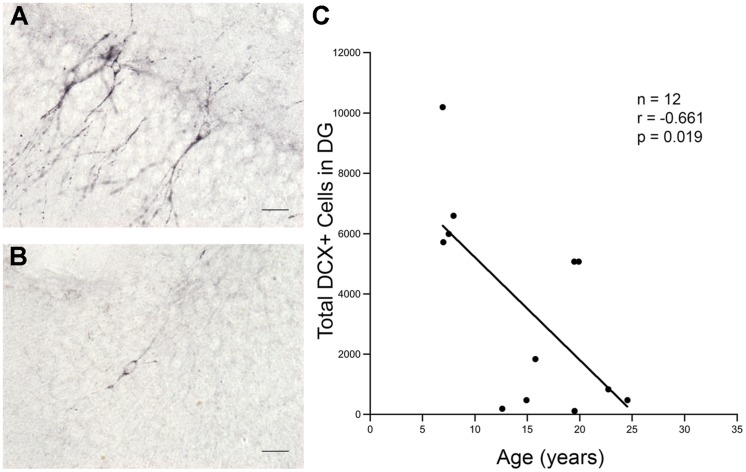
**Total number of DCX positive cells in the DG declines sharply with age.** More DCX positive cells are seen in the granule cell layer of the DG in young animals than in old animals. **(A)** DCX positive cells in a 7.9 years-old animal. **(B)** DCX positive cells in a 24.5 years-old animal. Scale bar for **(A,B)** = 20 μm. **(C)** There is a significant decline in the number of DCX positive cells present with increasing age.

Twelve monkeys aged 6.9–24.5 years (**Table 1**) were processed for the immature neuronal marker DCX. As shown in **Figures 2A,B**, DCX positive cells with features of immature neurons were seen in the GCL of the DG in both young and old monkeys. As shown in **Figure 2C**, stereological analysis showed a significant decrease in the number of DCX immunopositive cells with age (*r* = -0.661, *p* = 0.019).

### Newly Created Neurons Show Prolonged Maturation but Survive for Over a Year

**FIGURE 3:**
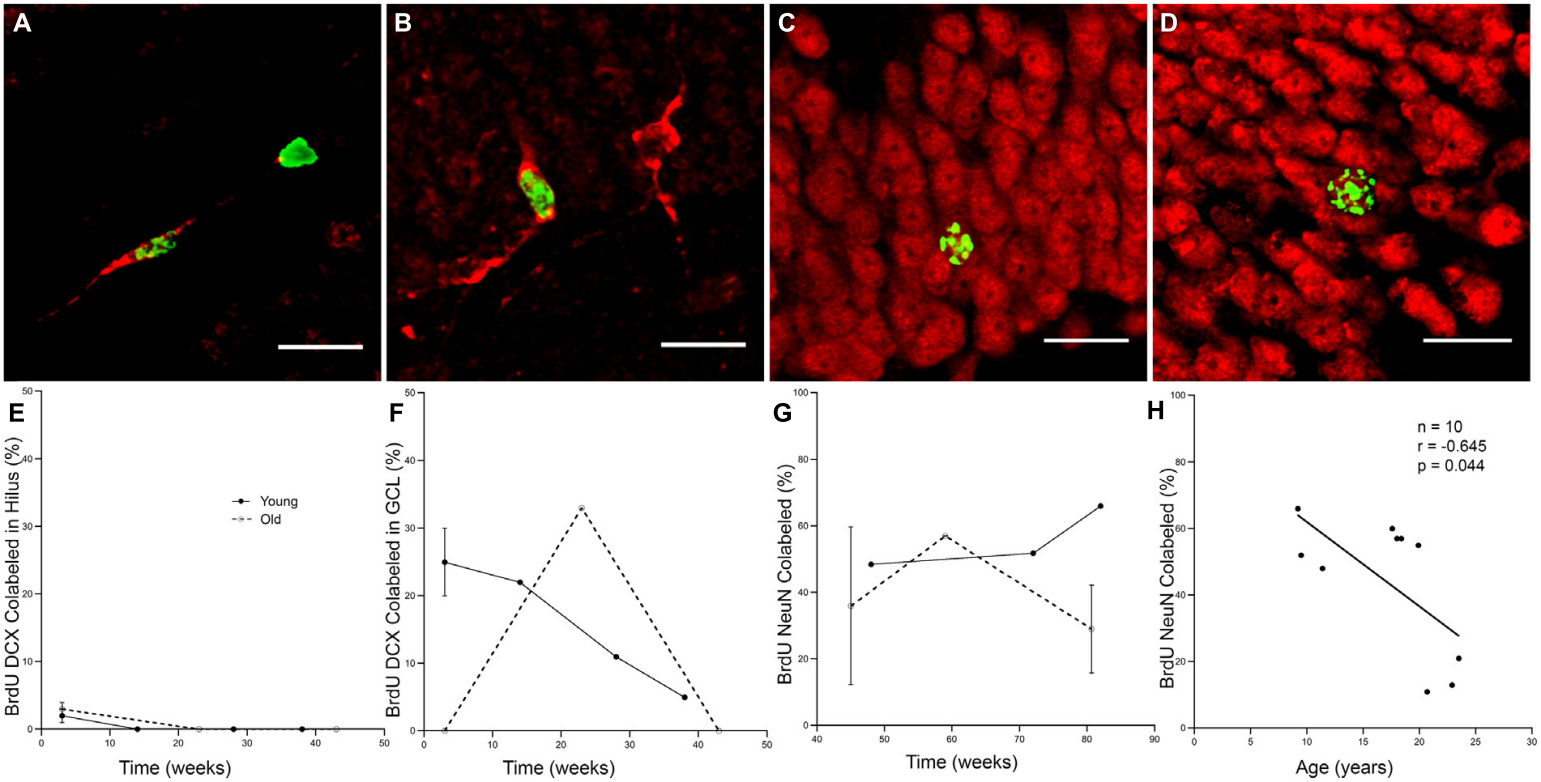
**Newly generated cells differentiate into mature neurons, however, the process may be delayed in aged animals. (A)** A BrdU (green) and DCX (red) double labeled cell in the hilus of the DG of a 6.9 years monkey at 3 weeks post-BrdU injection. **(B)** Most BrdU and DCX positive cells are seen in the GCL, as in this 8 years-old monkey with a 38-weeks survival time. **(C)** New mature neurons, as labeled with both BrdU (green) and NeuN (red) are seen in the GCL of animals with survival times longer than 1 year, as in this 9.2 years-old animal with an 82-weeks survival time. **(D)** Old animals also continue to have survival of new neurons as in this 19.9 yr old monkey with an 83-weeks post-BrdU survival time. Scale bars in **(A–D)** = 20 μm. **(E)** Immature neurons are sparse in the hilus, especially at longer survival times. **(F)** The majority of BrdU and DCX double labeled cells are found in the GCL. Whereas young animals average 25% BrdU positive cells express DCX, no immature neurons are seen in old animals at 3 weeks. With prolonged survival time in young animals, the percentage of BrdU positive DCX double labeled cells declines, yet in aged animals, BrdU and DCX double labeled cells are just beginning to be seen with longer survival times. **(G)** As survival times post-BrdU approach 1 year and beyond, both young and old animals show a fairly steady percentage of new mature neurons as seen by percentage of BrdU labeled and NeuN double labeled cells in the GCL. **(H)** In animals with post-BrdU survival times between 43 and 83 weeks, the percentage of BrdU and NeuN double labeled cells declines with age.

To determine how long it takes immature neurons to show mature phenotype and how long they can survive, 10 young and 12 old monkeys were injected with a single dose of BrdU and perfused at varying time points ranging from 3 to 83 weeks as shown in **Table 3**. Analysis of labeled cells revealed that young and old animals had BrdU positive cells that double-labeled with immature neuronal marker DCX, with the majority of the double-labeled cells being located in the GCL (**Figures 3A,B,E,F**). At 3 weeks, BrdU cells double-labeled with DCX were seen in young animals, but none were present in older animals (**Figures 3B,F**; *n* = 4). However, BrdU/DCX double-labeled cells were seen in an aged animal at 23 weeks, thus the integration process may be delayed in older animals. At BrdU time points of greater than 43 weeks, BrdU labeled cells double-labeled with mature neuronal marker NeuN were present in the GCL of both young and aged animals (**Figures 3C,D,G**). Although the oldest animals demonstrated newly generated cells that showed neuronal morphology at greater than 43 weeks, aged animals had consistently lower percentages of BrdU/NeuN double-labeled neurons (**Table 3**; **Figure 3H**; *r* = -0.645, *p* = 0.044).

### Increases in Granule Cell Numbers and Volume with Age

**FIGURE 4:**
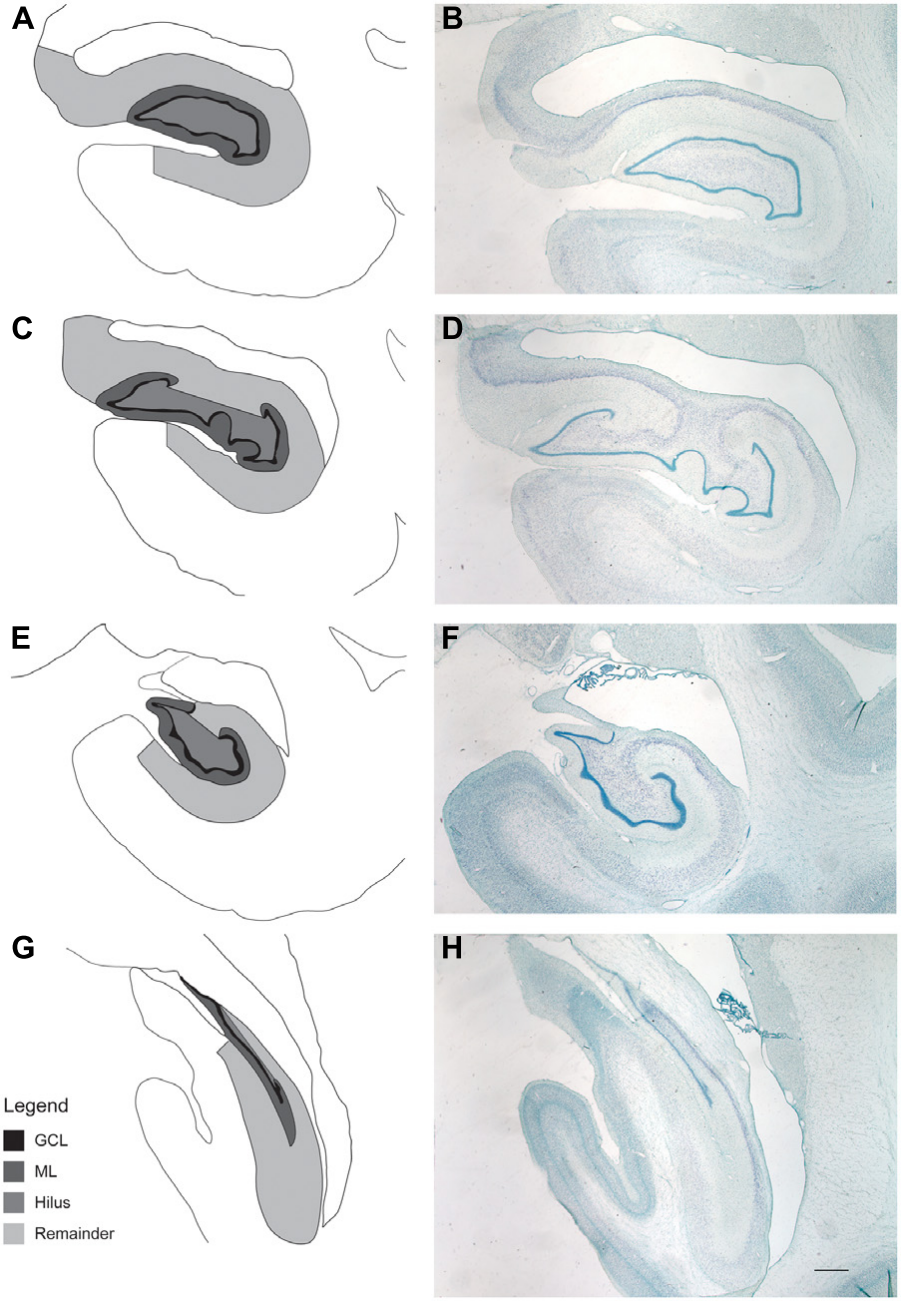
**Regions of interest for stereological evaluation of hippocampal cell numbers and volumes**. The entire rostral-caudal extent of the hippocampus was evaluated. Representative schematics **(A,C,E,G)** and photomicrographs **(B,D,F,H)** from rostral to caudal thionin stained sections are shown. Scale bar = 1 mm. The granule cell layer (GCL), molecular layer (ML), and hilus of the DG were analyzed separately and defined as outlined. For consistency of ROI, the hilus was defined as that area in between the blades of the granule cell layer. The “remainder of the hippocampus” included that area which represents CA3, CA2, and CA1, as outlined.

**FIGURE 5:**
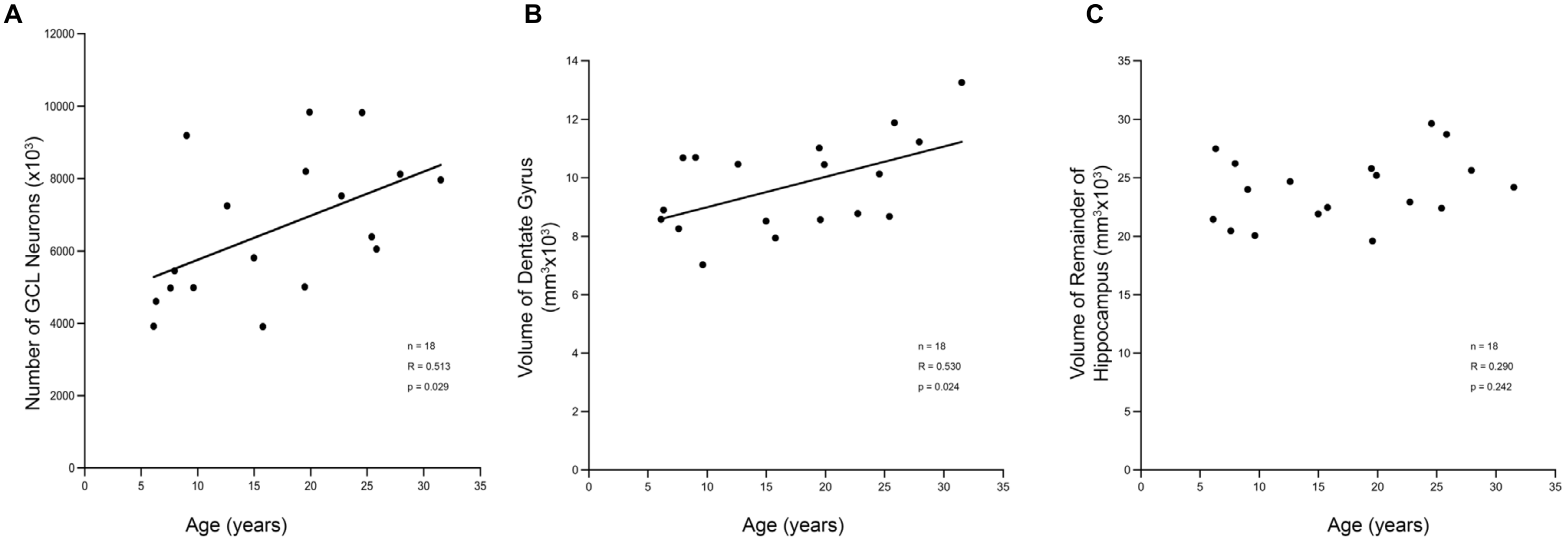
**Stereological analysis of DG shows increases with age. (A)** The estimated total number of granule cell neurons in the hippocampal DG increases over the lifespan of the rhesus monkey. This corresponds to a volume increase in the DG **(B)**. However, the remainder of the hippocampus, which includes non-neurogenic CA3, CA2, and CA1 does not show a corresponding increase with age **(C)**.

Stereology was used to determine whether ongoing adult neurogenesis leads to an increase in cell number with age. The total number of granule cells as well as the volume of the DG and the remainder of the hippocampus was examined in 18 male rhesus monkeys ranging from 6.1 to 31.5 years of age (**Table 1**), with ROI established for the GCL and the hilus of the DG (the polymorphic cell area within the blades of the GCL) as illustrated in **Figure 4**. Results demonstrate that granule cell numbers significantly increase over the lifespan of the rhesus monkey (*r* = 0.513, *p* = 0.029; **Figure 5A**). There was also an age-related increase in the volume of the total DG (*r* = 0.530, *p* = 0.024; **Figure 5B**), while the volume of the remainder of the hippocampus remained stable with age (*r* = 0.290, *p* = 0.242; **Figure 5C**).

### Some Age-Related Changes in Neurogenesis Correlate with Cognitive Decline

**FIGURE 6:**
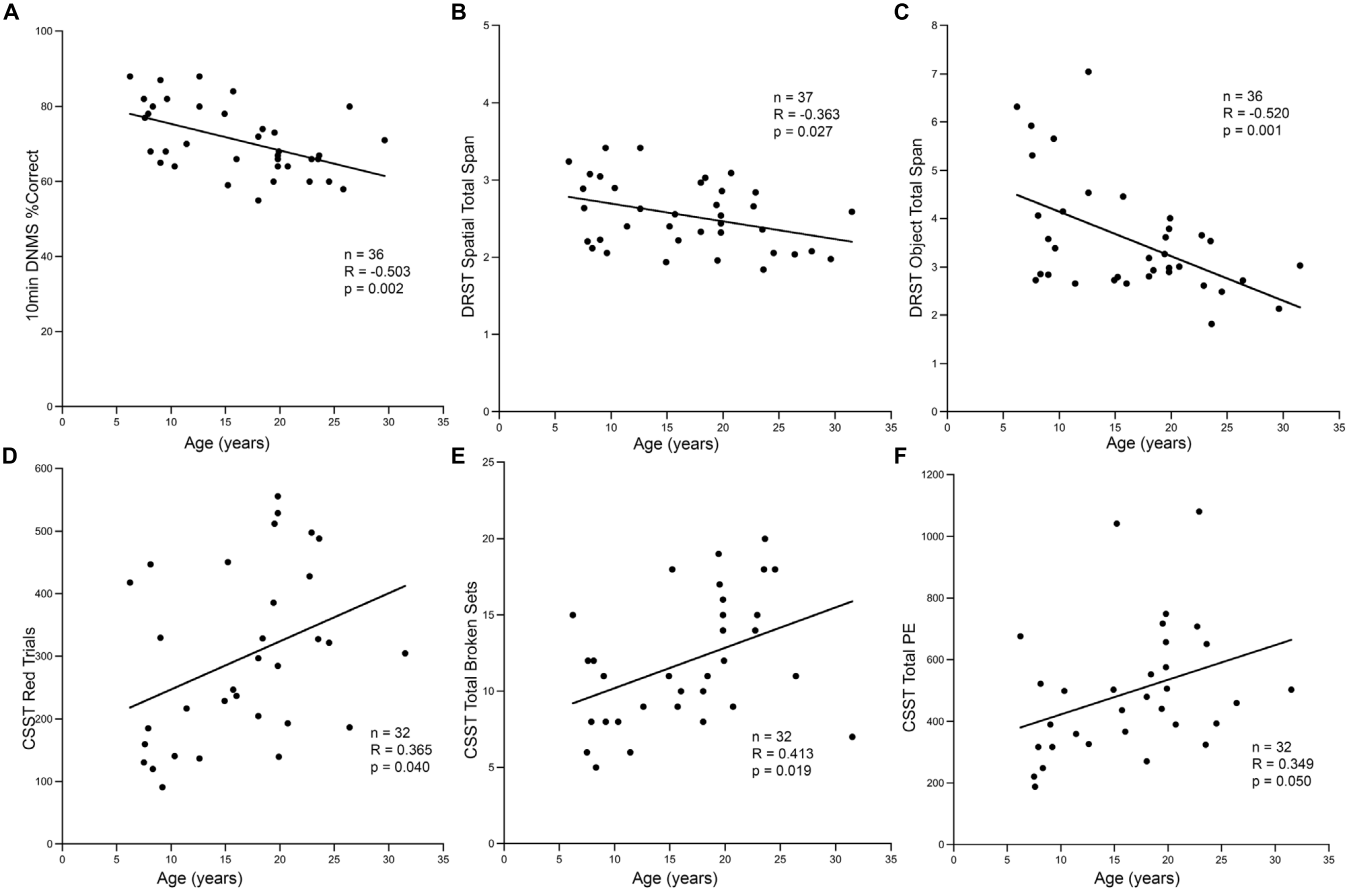
**Cognitive decline in aging animals. (A)** In the hippocampal dependent task DNMS with 10-min delay, the percentage of correct responses decreased with increasing age (*r* = -0.503, *p* = 0.002). A similar response was seen in the DRST task, both spatial and object conditions. The total span obtained in DRST object **(B)** and DRST spatial **(C)** was negatively correlated with age (*p* = 0.027, *p* = 0.001; respectively). **(D)** In CSST, the number of trials required to master initial abstraction increased with age (*r* = 0.365, *p* = 0.040). **(E)** The total number of broken sets (*r* = 0.413, *p* = 0.019) and **(F)** the total number of perseverative errors also showed a positive correlation with increasing age (*r* = 0.349, *p* = 0.050).

Consistent with other studies on cognitive aging in rhesus monkeys, we found an age-related decline in cognitive function as illustrated in **Figure 6**. Aged animals had fewer correct responses on the 10-min delay DNMS task, and had shorter spans on both DRST spatial and object tasks (**Figures 6A–C**). Aged animals also required more trials to reach criterion on the initial abstraction of CSST and had an increased total number of broken sets and more perseverative errors (**Figures 6D–F**). When CSST analysis was limited to the cohort of animals that had histological results, the relationships between age and trials to initial abstraction (*r* = 0.751, *p* = 0.001), total broken sets (*r* = 0.848, *p* < 0.001), and total preservative errors (*r* = 0.674, *p* = 0.006) were all significant.

**FIGURE 7:**
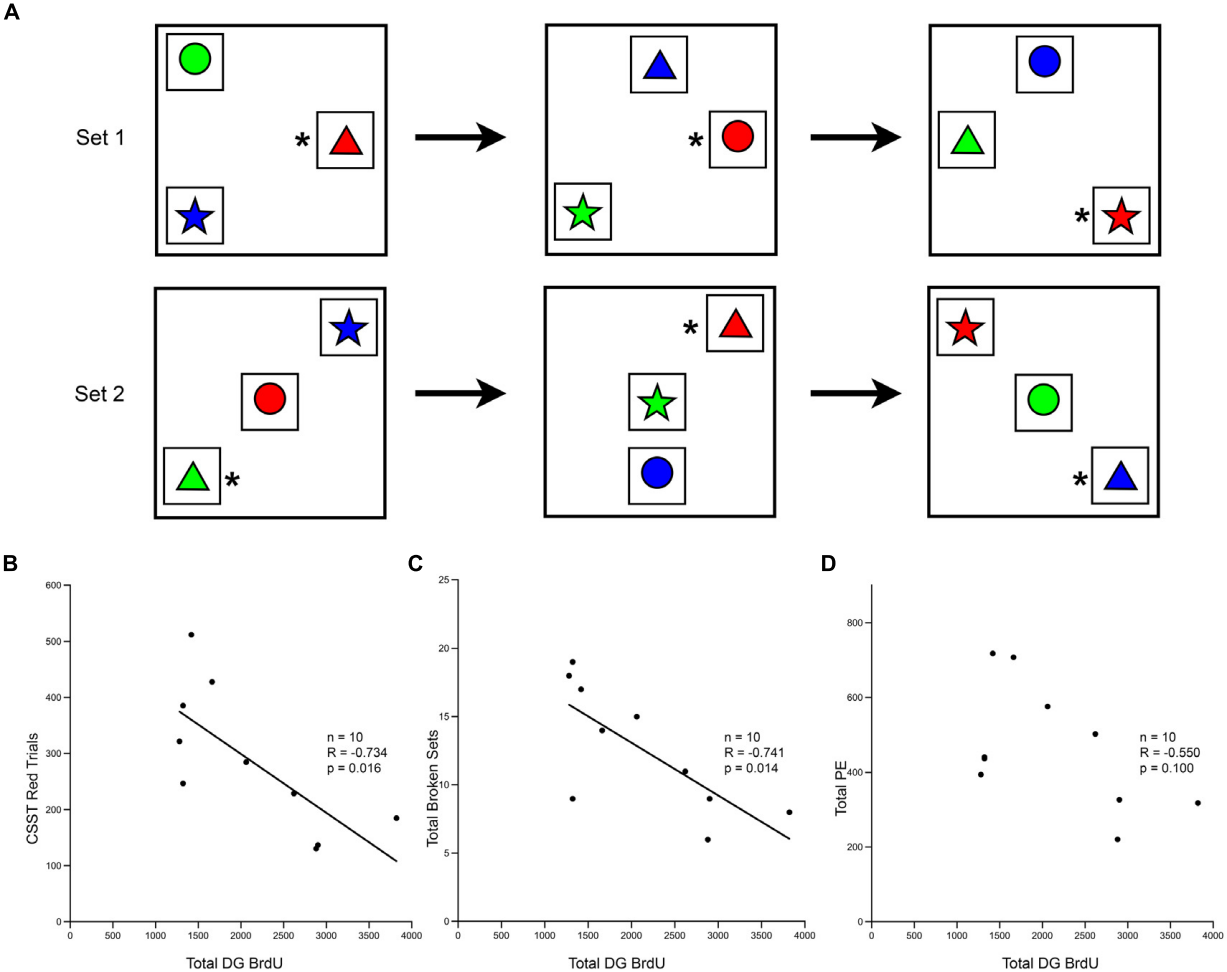
**Short-term survival of newly generated cells shows correlation with the cognitive task CSST. (A)** Schematic of the CSST task in which each box represents a trial; the asterisk represents the correct answer. Animals first learn that “red” is correct, as shown in the first row of trials. After ten consecutive correct responses, the correct response shifts to the new concept of “triangle”, and a new set of trials begins, as illustrated. **(B)** The total number of BrdU positive cells in the DG after a 3 weeks survival was negatively correlated with the number of trials needed for initial abstraction (*r* = -0.737, *p* = 0.015). **(C)** The total number of broken sets was also negatively correlated with BrdU number (*r* = -0.741, *p* = 0.014). **(D)** The total number of perseverative errors showed a trend, but was not significantly correlated with the total number of BrdU cells present in the DG at 3 weeks (*r* = -0.550, *p* = 0.100).

Correlational analysis between these CSST cognitive measures and measures of cell proliferation and neurogenesis, revealed a significant association with performance on the CSST (**Figure 7A**). The total number of BrdU positive cells in the DG after a 3 weeks survival correlated strongly with the initial learning of the CSST (**Figure 7B**; *r* = -0.734, *p* = 0.016). The total number of broken sets also showed an inverse relationship with age and BrdU (**Figure 7C**; *r* = -0.741, *p* = 0.014). While the total number of perseverative errors was strongly correlated with age in the total behavioral cohort, the relationship to BrdU cell number only approached significance (**Figure 7D**; *r* = -0.550, *p* = 0.100).

Further analysis using stepwise multiple regression among age, BrdU number at 3 weeks survival, and CSST results, revealed that age was the strongest predictor of CSST performance (*p* = 0.009). Whereas age accounted for 59% of the variance in CSST results, total BrdU numbers accounted for only 6% of the variance, which was not significant (*p* = 0.529). No significant correlations were found between any of the other histological measures (e.g., DCX cell number, or total number of DG cells) and the other cognitive tasks (data not shown).

## Discussion

### Summary of Results

Consistent with previous data showing an age-related decline in hippocampal neurogenesis, this study demonstrates a 68% decline in Ki-67 labeled proliferating cells, and a 53% decline in BrdU labeled S-phase cells. In addition to these declines, multiple labeling with markers of both immature and mature neurons suggests that maturation of new neurons is prolonged in the aged monkey, but approaches similar percentages of mature new neurons with an average of 48% of BrdU labeled cells co-localized with NeuN after 48 weeks in the young animals, and 36% of BrdU labeled cells co-localized with NeuN after 45 weeks in aged animals. The quantitative 41% increase in the number of granule cells with age suggests that neurogenesis results in a gradual addition of new neurons throughout the lifespan. Finally, while there was no significant correlations between markers of adult neurogenesis and a global measure of age-related decline in learning it remains to be determined if tasks more specific to the DG might reveal a relationship.

### Declines in Neurogenesis with Age

Declines in hippocampal neurogenesis with aging have been well-established in rodents (Kuhn et al., 1996; Seki, 2002; McDonald and Wojtowicz, 2005; Rao et al., 2005, 2006; Morgenstern et al., 2008). McDonald and Wojtowicz (2005) demonstrated a 92% reduction in the number of cells labeled by BrdU between rats at 38 days of age compared to rats at 12 months of age. Similarly, Rao et al. (2006) reported a decline in BrdU labeling of 80%, and a corresponding decline in Ki-67 labeling of 85% between young and old Fischer 344 rats. Studies in primates addressing changes in neurogenesis with age are more limited. In a study of seven cynomologus monkeys, Aizawa et al. (2009) demonstrated an age-related decrease in Ki-67 labeled cells of 80%. Leuner et al. (2007) used a BrdU paradigm similar to that used here (single injection of 200 mg/kg with a survival time of 3 weeks) and reported an age-related decline of BrdU labeled cells in the GCL of about 47% comparing young and middle-aged common marmosets. The present data in rhesus monkeys shows declines that are far less severe than those reported in rodents with declines across the life span (7–25 years of age) of 68% in Ki-67 labeling of proliferating cells and 53% in BrdU (3-weeks survival) labeling of DNA replication (**Figure 1**). Similarly, in humans, carbon dating based on isotopes generated between 1955 and 1963 by above ground nuclear testing reported that hippocampal neurogenesis continues into old age without significant decline (Spalding et al., 2013). Overall, published studies and the present data confirm that cell proliferation declines with aging, but that this decline may be less severe than that observed in rodents.

### Maturation of New Neurons is Prolonged in Primates

Studies in aged rodents report a delay in neuronal maturation. In rats, Rao et al. (2005, 2006) reported a delay in migration and maturation of newly created neurons with aging. Despite the delay, they found that a similar percentage of cells eventually become new neurons in young and aged rats (∼73–76%). Interestingly, in the present study, at 3 weeks after injection of BrdU, immature neurons (double labeled with BrdU and DCX) were present in young animals (*n* = 4) but were absent in aged animals (*n* = 4). However, maturing adult generated neurons (BrdU+ and DCX+) were present 23 weeks after BrdU injection, our next available time point. This suggests there is an age-related delay in maturation of newly generated neurons in monkeys. It is also interesting that in aged monkeys just under 1 year (∼45 weeks) after BrdU injection, 36% of BrdU labeled cells were mature neurons as indicated by BrdU and NeuN double labeling, only slightly less than the 48% of BrdU neurons that were NeuN positive after∼48 weeks in a young animal. These data provide evidence that similar to the rodent data, newly generated cells eventually become mature neurons at comparable rates in both young and aged monkeys (**Table 3** and **Figure 3**). However, since our available time points after BrdU injection are not continuous, the precise delay in maturation occurring in aging monkeys cannot be determined.

### Adult Generated Neurons may Account for Increased Granule Cell Numbers

Classic studies of adult neurogenesis in rodents reported that newly generated neurons led to cumulative increases in granule cell number (Bayer, 1982; Crespo et al., 1986). However, these studies examined only juvenile and adult animals and did not explore the potential for the ongoing addition of neurons over the entire adult life span. In contrast, more recent studies in aged laboratory rodents failed to detect an age-related increase in granule cell number (Rapp and Gallagher, 1996; Rasmussen et al., 1996; Kempermann et al., 1998; Merrill et al., 2003). But studies comparing young and aged wild bank voles and wild wood mice found an increase in the total number of hippocampal granule cells of 30–40% (Amrein et al., 2004). In a study of young rhesus monkeys, Jabès et al. (2010) report a large increase in neuron number between newborn and 3 months, and a stable number of granule cell neurons in 5–10 years-old monkeys, but did not examine neuron number in animals older than 10. In contrast, Keuker et al. (2003) used stereology to count hippocampal neuron number in a study of rhesus monkeys from newborn to 4 years of age compared to aged subjects from 18 to 31 years of age, and reported no age-related loss of granule cells. Interestingly, their data table shows that there were 7.8 million neurons in the DG GCL of the aged animals, but only 5.6 million in their young group, a difference that approached significance at *p* = 0.06 despite the small sample size (*n* = 8 young and 5 old). These granule cell numbers constitute an increase over the life span of about 39%, which is almost identical to the 41% increase reported in the present study.

The increase in granule cell numbers over the life span of the monkey suggested by our data and that of Keuker et al. (2003) together with the increase over the lifespan reported by Amrein et al. (2004) for wild rodents, raises the question of what might be different for laboratory rodents where no increase was found. In this regard, the issue of “enriched” versus impoverished environment is pertinent. It is well-established that learning and environmental enrichment facilitates adult neurogenesis (Kempermann et al., 1997; Tashiro et al., 2007; Kobilo et al., 2011). In this regard, one can speculate that the environment for wild rodents is likely to be highly enriched compared to caged laboratory rodents. Similarly, it is important to note that the majority of the monkeys studied here were from the Yerkes National Primate Research Center, where they lived most of their lives in outdoor social groups in large corrals, an enriched environment compared to isolated cages of laboratory rodents. Even after entry into our aging study, these monkeys received considerable enrichment in the form of daily cognitive behavioral testing. These forms of enrichment in our monkeys may explain the presence of an age-related increase in the numbers of DG neurons over the lifespan. Additionally, it is interesting that studies of granule cell numbers in human brain (West, 1993; Simic et al., 1997; Harding et al., 1998) have not detected an increase in granule cell numbers, but have documented large individual differences in granule cell numbers. While humans are unlikely to experience the impoverished environments of laboratory rodents, human environments are likely to be much more variable than environmental conditions in national primate centers, perhaps contributing to the large variability in granule cell numbers.

### Relationship between Adult Neurogenesis and Cognitive Decline

If adult neurons do survive and integrate into the DG, the question is whether they contribute to normal cognitive performance, and whether the reduction across the lifespan contributes to age-related cognitive decline. Interestingly, multiple studies conducted in rodents, have reported that adult generated neurons in the GCL are important for tasks such as pattern separation, concordant with the role of the DG in minimizing interference between overlapping spatial or contextual information. Ablation of neurogenesis results in impaired performance on spatial discrimination tasks when stimuli are presented with little spatial separation (Clelland et al., 2009). Additionally, the reduction of adult neurogenesis has been suggested to impair the ability of the DG to discriminate between temporal contexts, which are important for the encoding of episodic memories (Rangel et al., 2014). Moreover, it has been shown that enhancement of neurogenesis improves performance on pattern separation tasks (Sahay et al., 2011).

Aizawa et al. (2009) evaluated learning performance in young and aged cynomologus monkeys using the visual pattern discrimination task. In young animals, their results demonstrated a positive correlation between levels of Ki-67 cells and performance on the task, with further evidence that monkeys with good learning performance exhibited higher levels of Ki-67 cells compared to animals with impaired performance. In aged animals, this trend was not observed as there was greater individual variability of learning performance.

In the present study, the correlations with learning were seen with BrdU labeled cells that had a 3-weeks survival period, at which time mature neuronal phenotype is not expressed. Throughout the maturation process, new neurons progress through different stages of maturation characterized by distinct morphological and physiological properties. As a result, within the DG, the granule cell population includes adult generated neurons of different ages, and hence at different stages of maturation (Deng et al., 2010). At the early stages of maturation, new neurons have limited branching and few connections with other cells (Ngwenya et al., 2006). As maturation proceeds, dendritic spines form (Ngwenya et al., 2006) and mossy fiber axons continue to extend toward CA3 cells where they begin to make synaptic contacts (Zhao et al., 2008). At about 4 weeks in mice, new neurons are highly excitable, exhibit a low threshold and high spiking activity (Ambrogini et al., 2004; Mongiat et al., 2009; Marin-Burgin et al., 2012). By 8 weeks, a mature morphological phenotype is established, hyper-excitability is diminished and threshold levels for spiking are similar to mature granule cells (Mongiat et al., 2009), suggesting that these adult generated neurons have become fully functional granule cells able to contribute to hippocampal information processing. The maturation timeline in the macaque is at least 4 times longer (3–6 months; Ngwenya et al., 2006; Kohler et al., 2011) than the rodent (3–6 weeks). Consequently, the present data on BrdU numbers at 3 weeks of maturation may not sufficiently reflect functional integration into the DG circuitry. Similarly, given the prolonged expression of DCX in the monkey, neurons expressing DCX are less than 6 months of age (Kohler et al., 2011) and are also unlikely to reflect functional integration.

In assessing possible relationships between neurogenesis and behavior, the only task that showed a significant correlation with indices of neurogenesis was initial learning on the CSST (Moore et al., 2005, 2009). This initial abstraction of CSST is similar to the visual pattern discrimination task used in Aizawa et al. (2009) as both tasks require learning a complex, visually distinct category. Moreover, the initial learning on the CSST is likely a hippocampal function and it correlated with neurogenesis indices, while the subsequent CSST shifts, represented by perseverative errors are thought to be dorsolateral prefrontal functions (Moore et al., 2009), and did not significantly correlate. It seems likely that a monkey version of the pattern separation task, specifically designed to test DG function as seen in rodents and humans (Clelland et al., 2009; Yassa et al., 2011), might be more sensitive to age-related reductions in neurogenesis. The CSST is the task in our battery that is operationally closest to a pattern separation task due to the visual similarity and spatial proximity of the objects that have to be chosen. Moreover, it is by far the hardest to “learn” compared to the other tests (**Figure 7A**). Nevertheless, it is possible that a task specifically designed as a pattern separation task for monkeys would be a better assay for the relationship of cognition to age-related decline in neurogenesis.

While the number of BrdU labeled cells showed some correlation with performance on CSST, careful statistical analysis identified age as a stronger predictor of performance. This suggests that although neurogenic proliferation may be important in learning, other factors that change with the age likely also contribute to age-related cognitive impairments, i.e., cognitive aging is likely multifactorial. For example, even though DNMS and DRST are sensitive experimental damage to the hippocampus, in the monkey, the hippocampus is directly connected to multiple brain regions (e.g., amygdala, EC, cingulate cortex, prefrontal cortex; Rosene and Van Hoesen, 1977; Saunders and Rosene, 1988; Aggleton et al., 2015), structures that likely contribute to performance on these tasks. Moreover, published data demonstrates that similar to hippocampal lesions, damage to prefrontal cortex in monkeys also impairs performance on DNMS (Moore et al., 2012). The CSST is primarily considered a task of prefrontal cortex function, but given the multiple aspects of cognition assessed with this test (e.g., acquisition, learning, set-shifting, response suppression), it is likely that other cortical areas such as the DG, hippocampus, and EC, are also involved in CSST performance. Thus the age-related cognitive deficits detected in our behavioral test battery likely reflect global changes within the aging brain with declines in neurogenesis only one of many factors affecting cognitive aging.

## Conclusion

This study demonstrates that neurogenesis in the monkey DG continues into old age, but the rate of neuron generation declines, and neuronal maturation is delayed with age. Despite the diminished generation and delayed maturation, ∼50% of newly generated cells continue to express mature neuronal markers at greater than 1 year in all but the very oldest of monkeys (i.e., those over 20 years of age) where the rate may be reduced to 20%. Analysis of total DG granule cell numbers across the life span shows that a proportion of these new neurons survive and add to the complement of neurons in the DG where there is a modest but steady increase in number with age. While the number of BrdU positive cells labeled after a 3 weeks survival significantly correlates with performance on the initial category learning in a set-shifting task, age alone is the best predictor of performance, suggesting that multiple factors underlie age-related cognitive impairment. Overall, although neurogenesis declines with age in the rhesus monkey, many new neurons continue to be generated and likely persist in sufficient numbers to contribute to the functioning of the DG. Based on these results, it will be important to determine how newly generated dentate granule cells function in the context of new learning, as conditions which delay or perturb adult neurogenesis such as aging, traumatic brain injury, and various stressors including psychiatric conditions may benefit from interventions to enhance new neuron generation and functional integration.

## Author Contributions

LN and NH contributed equally to data analysis and writing the manuscript. LN and DR contributed to the conception and design of the work. TM contributed to the conception and creation of the CSST test and the acquisition and interpretation of the behavioral data. LN contributed to the acquisition, analysis, and interpretation of the BrdU, Ki-67, and hippocampal stereological data. NH contributed to the acquisition and analysis of the DCX immunohistochemical data, acquired the BrdU and DCX fluorescence data, and contributed to the analysis and interpretation of results. YS performed the BrdU and NeuN fluorescence data and contributed to the analysis and interpretation of the results. All authors played a role in drafting and revising the manuscript, approved the final version, and agree to be accountable for all aspects of the work.

## Conflict of Interest Statement

The authors declare that the research was conducted in the absence of any commercial or financial relationships that could be construed as a potential conflict of interest.

## Abbreviations

BrdU: bromodeoxyuridine
CSST: category set shifting task
DCX: doublecortin
DG: dentate gyrus
dDG: dorsal dentate gyrus
DNMS: delayed non-match to sample task
DRST: delayed recognition span task
NeuN: neuronal nuclei
NHP: non-human primate
ROI: region of interest.
